# Hypoxia-inducible factor-1*α* expression in the gastric carcinogenesis sequence and its prognostic role in gastric and gastro-oesophageal adenocarcinomas

**DOI:** 10.1038/sj.bjc.6603524

**Published:** 2006-12-19

**Authors:** E A Griffiths, S A Pritchard, H R Valentine, N Whitchelo, P W Bishop, M P Ebert, P M Price, I M Welch, C M L West

**Affiliations:** 1 Academic Radiation Oncology, Division of Cancer Studies, The University of Manchester, Christie Hospital, Wilmslow Road, Manchester M20 4BX, UK; 2 Department of Gastrointestinal Surgery, South Manchester, University Hospitals NHS Trust, Manchester M23 9LT, UK; 3 Department of Histopathology, South Manchester, University Hospitals NHS Trust, Manchester M23 9LT, UK; 4 Department of Medicine II, Klinikum rechts der Isar, Technical University Munich, Munich D-81675, Germany

**Keywords:** gastric cancer, gastro-oesophageal junction tumours, HIF-1*α*

## Abstract

Hypoxia-inducible factor-1 (HIF-1)*α* expression was studied in the gastric carcinogenesis sequence and as a prognostic factor in surgically resected gastric and gastro-oesophageal junction tumours. Protein expression was examined using immunohistochemistry on formalin-fixed biopsies of normal mucosa (*n*=20), *Helicobacter pylori* associated gastritis (*n*=24), intestinal metaplasia (*n*=24), dysplasia (*n*=12) and intestinal (*n*=19) and diffuse (*n*=21) adenocarcinoma. The relationship between HIF-1*α* expression and prognosis was assessed in resection specimens from 177 patients with gastric and gastro-oesophageal junction adenocarcinoma. Hypoxia-inducible factor-1*α* expression was not observed in normal gastric mucosa but increased in density (*P*<0.01) and intensity (*P*<0.01) with progression from *H. pylori*-associated gastritis, intestinal metaplasia, dysplasia to adenocarcinoma. The pattern of staining in the resection specimens was focally positive in 49 (28%) and at the invasive tumour edge in 41 (23%). Invasive edge expression was associated with lymph node metastases (*P*=0.034), advanced TNM stage (*P*=0.001) and was an adverse prognostic factor for cancer-specific survival (*P*=0.019). In univariate analysis and in comparison with tumours not expressing HIF-1*α*, invasive edge staining was associated with a hazard ratio of 1.6 (95% CI 1.0−2.5) and focally positive staining a hazard ratio of 0.7 (95% CI 0.5−1.2). Hypoxia-inducible factor-1*α* lost prognostic significance in multivariate analysis. The results suggest HIF-1*α* is involved in gastric carcinogenesis and disease progression, but is only a weak prognostic factor for survival.

Tumour hypoxia is now recognised as a key factor driving the development of malignancy, and the master regulatory protein in the response of cells to changing oxygen levels is hypoxia-inducible factor-1 (HIF-1). Hypoxia-inducible factor-1 consists of *α* and *β*-subunits which are both members of the helix-loop-helix family of transcription factors (Semenza, 2003). The *β*-subunit is constitutively expressed and its activity is controlled in an oxygen-independent manner. The *α*-subunit is ubiquitinated and degraded in normoxia, but stabilised in hypoxia. In the hypoxic environment, HIF-1*α* dimerises with HIF-1*β* and binds to hypoxia-responsive elements (HRE) within the nucleus. A wide variety of genes, including VEGF, Glut-1, CA9, erythropoietin and iNOS are known to have HREs and are activated by HIF-1*α*. Non-hypoxic activators are now known to include growth factors, cytokines, tumour suppressor genes, oncogenes, viruses and bacteria (Griffiths *et al*, 2005).

Hypoxia-inducible factor-1*α* activity appears to be a very early event in carcinogenesis and the protein is expressed before histological evidence of angiogenesis or invasion (Zhong *et al*, 1999). Zhong *et al* (1999) first observed HIF-1*α* expression in a few cases of pre-malignant breast, prostatic and colonic tissue. Subsequent studies with greater numbers of patients showed that HIF-1*α* expression is involved, and progressively increased expression has been observed in the pre-malignant phases and developmental steps of breast (Bos *et al*, 2001), skin (Costa *et al*, 2001) and cervical (Acs *et al*, 2003) cancer. In prostatic carcinoma, HIF-1*α* expression was highly expressed in the precursor lesion, prostatic intra-epithelial neoplasia (Zhong *et al*, 2004).

Histologically, gastric cancers are classified into two types: diffuse and intestinal (Lauren, 1965). For the development of intestinal gastric cancer, a multistep process involving a progressive cascade of molecular and morphological changes has been proposed by Correa (2004). Diffuse tumours have no known pre-malignant precursor lesions. For both types of tumour the carcinogenesis process is believed to be initiated by *Helicobacter pylori* infection and the risk of gastric cancer development has been related to *H. pylori* strain type, other environmental factors, host genetic factors and immune-related polymorphisms (Peek and Blaser, 2002; Nardone *et al*, 2004). Although *H. pylori* can directly or indirectly alter intracellular signalling in the gastric mucosa, leading to increased proliferation and apoptosis, host inflammation appears to be of key importance in tumour development. Additional mutational genetic and epigenetic events in the tumour or neighbouring cells lead to progressive tumour development. These events and the inflammatory process are believed to adaptively transform *H. pylori*-induced chronic gastritis into intestinal metaplasia, epithelial dysplasia and finally intestinal-type carcinoma. Hypoxia-inducible factor-1*α* has been implicated in this process: a cell line study has shown that reactive oxygen species (ROS), produced by *H. pylori,* stabilise HIF-1*α*, leading to increased expression (Park *et al*, 2003).

Hypoxia-inducible factor-1*α* is expressed in a variety of human cancers (Zhong *et al*, 1999), and has been linked with a poor prognosis in patients who received radiotherapy (Aebersold *et al*, 2001), chemotherapy (Sohda *et al*, 2004) or surgery (Kurokawa *et al*, 2003). As such, there is currently interest in the use of HIF-1*α* inhibition as a cancer therapeutic strategy. Two studies revealed encouraging results in murine models of gastric cancer (Yeo *et al*, 2003; Stoeltzing *et al*, 2004). They used either pharmacological or genetic inhibition of HIF-1*α*, which resulted in dramatic effects on tumour vascularisation and reduced growth of xenografts derived from human gastric cancer cells. However, studies of HIF-1*α* expression have been conflicting in several tumour subsites, including cervical, lung and ovarian cancer. Some studies have related high HIF-1*α* expression with an improved prognosis (Volm and Koomagi, 2000; Beasley *et al*, 2002). Although HIF-1*α* expression was associated with a poor prognosis in gastrointestinal stromal tumours of the stomach (Takahashi *et al*, 2003; Chen *et al*, 2005b), there are conflicting prognostic data in patients with gastric adenocarcinoma (Mizokami *et al*, 2006; Sumiyoshi *et al*, 2006; Urano *et al*, 2006). These studies involved Japanese patients, where there is a predilection for distal gastric cancer and relative lack of gastro-oesophageal junction tumours. Therefore, the prognostic effect of HIF-1*α* expression in tumour locations more representative of the UK population remains unknown. Also HIF-1*α* expression has yet to be studied in a gastric carcinogenesis model.

Therefore, the aims of this study were to investigate HIF-1*α* expression by immunohistochemistry in the pre-malignant and malignant gastric tissue progression sequence, and to assess the prognostic value of HIF-1*α* expression in surgically treated gastric and gastro-oesophageal cancer patients.

## MATERIALS AND METHODS

### Human tissue specimens

Tissue specimens were obtained from the histopathology archive of the Department of Histopathology, South Manchester University Hospitals NHS Trust. The study was approved by the South Manchester Ethics Committee.

### Gastric biopsies specimens

Formalin-fixed endoscopic gastric biopsy samples of normal gastric mucosa (*n*=20), *H. pylori* associated gastritis (*n*=20), intestinal metaplasia (*n*=20), epithelial dysplasia (*n*=12) and intestinal (*n*=19) and diffuse (*n*=21) type gastric adenocarcinoma were obtained. Endoscopy reports were obtained to ensure the correct biopsy location. Four of the biopsies had both *H. pylori* infected mucosa and intestinal metaplasia tissue. The haematoxylin and eosin slides were reassessed by a consultant pathologist (SP) to ensure correct classification. All cases of *H. pylori*-associated gastritis showed significant numbers of organisms. The epithelial dysplasia group were classified as low (*n*=6) or high (*n*=6) grade. Intestinal metaplasia was present in six out of the 12 dysplasia biopsies.

### Surgically treated patients

A retrospectively compiled database was established of 251 consecutive patients with primary gastric and gastro-oesophageal junction tumours who underwent surgery at the South Manchester University Hospitals NHS Trust between 1995 and 2004. The Siewert classification was used to classify gastro-oesophageal junction tumours (Siewert and Stein, 1998). Patients who had either Siewert Type I gastro-oesophageal tumours (*n*=22), neo-adjuvant therapy (*n*=31), emergency surgery (*n*=1), completion gastrectomy (*n*=6) or died after surgery (*n*=25) were excluded from the study. The study group therefore comprised 177 patients (125 males) with a median age of 68 (range 49–85) years. There were 76 Siewert type II, 21 type III gastro-oesophageal junction tumours and 80 non-cardia gastric cancers. Patients underwent either partial or subtotal gastrectomies (*n*=45), total gastrectomy (*n*=44), proximal gastrectomy (*n*=4) or oesophago-gastrectomy (*n*=84). Selected patients underwent additional surgical resection of the spleen (*n*=21) and spleen with distal pancreas (*n*=5). One hundred and thirteen patients (64%) underwent a potentially curative resection (R0 resection), defined as complete macroscopic and microscopic removal of the tumour on intraoperative assessment and subsequent histopathological evaluation. Fifty-four patients (31%) had residual microscopic disease (R1 resection), whereas 10 patients (6%) had residual macroscopic disease (R2 resection). After surgery, patients were followed in the surgical outpatient clinic. Hospital notes of the patients were reviewed and, if necessary, the local cancer registry or patient's general practitioner were contacted to complete case follow-up.

### Immunohistochemical staining of HIF-1*α*

The best tissue section for immunohistochemistry was selected and the corresponding formalin-fixed, paraffin-embedded resection specimen obtained. As deterioration in immunohistochemical staining occurs in stored sections (Bertheau *et al*, 1998; Olapade-Olaopa *et al*, 2001), specimens were stained within 2 months of cutting. Immunohistochemical detection of HIF-1*α* was performed using the Tyramide Signal Amplification System (NEN Life Sciences, Boston), which is based on a streptavidin–biotin–horseradish peroxidase complex formation. Sections 4 *μ*m thick were deparaffinised and the antigen retrieved by microwaving in 10 mM citrate buffer (pH 6.0) for 25 min followed by blocking steps according to the manufacturer's protocol. Mouse monoclonal antibody (610958, BD Biosciences, diluted 1 : 100) was applied and the slides incubated overnight at 4°C. The secondary antibody, biotinylated rabbit anti-mouse (DakoCytomation, Denmark), was applied with additional blocking precautions employed to minimise the amplification of nonspecific background (Kim *et al*, 2003). The antibody was visualised using diaminobenzidine (DakoCytomation, Denmark) and the sections counterstained with haematoxylin, dehydrated and mounted. Substitution of the primary antibody with the identical concentration of mouse immunoglobulins IgG1 (DakoCytomation, Denmark) served as negative controls. Batch-to-batch variation was assessed by choosing two sections showing high and low HIF-1*α* expression and running additional sections from these biopsies with each batch.

### Assessment of HIF-1*α* staining in the tissue sections

Only tumour nuclear HIF-1*α* staining was scored using a method modified from the literature that was previously used on gastric tissue (Ito *et al*, 2003). The scoring system was as follows: 0, no nuclear staining; 1, <2% nuclear staining; 2, 2–10% nuclear staining; 3, 10–29% nuclear staining; and 4, >30% nuclear staining. Nuclear staining intensity in the gastric biopsies was scored as weak, moderate or strong. Scoring was performed in a double-blind manner by two independent investigators (SP, EAG). Any disagreement was resolved by discussion to obtain a final score.

### Statistics

The non-parametric Jonckheere–Terpstra test was used to identify ordered differences among the biopsy categories in the gastric carcinogenesis sequence. With this test, the null hypothesis is that the distribution does not differ across ordered categories. The *χ*^2^-test was used to correlate HIF-1*α* expression and the various clinical and pathological characteristics of the patients studied. Survival time was measured as the time from the date of surgery until death or last follow-up appointment. Overall survival and cancer-specific survival were used as end points. Univariate survival analyses were performed using the Kaplan–Meier method. Factors were compared using the Cox proportional hazards model and log-rank tests. Multivariate survival analysis was performed on factors which achieved statistical significance (*P*<0.05) in univariate analysis, using the Cox proportional hazards model to identify independent predictors of survival.

## RESULTS

### Expression of HIF-1*α* in gastric biopsy specimens

**Figure 1:**
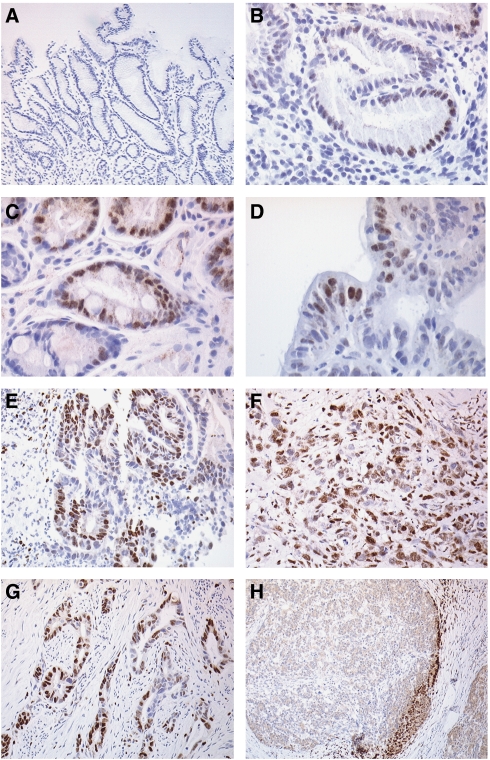
Photomicrographs of HIF-1*α* immunohistochemistry in the gastric cancer progression sequence showing no staining in normal mucosa (**A**), weak nuclear staining in mucosal cells in *H. pylori* gastritis (**B**), moderate staining in intestinal metaplastia (**C**), distinct nuclear staining in high grade dysplasia (**D**), and strong staining in well (**E**) and poorly (**F**) differentiated intestinal adenocarcinoma (**E**) and revealing moderate HIF-1*α* staining; (**D**) High grade dysplasia showing distinct nuclear HIF-1*α* staining. Well (**E**) and poorly (**F**) differentiated intestinal adenocarcinoma showing distinct strong nuclear HIF-1*α* staining. Photomicrographs of HIF-1*α* immunohistochemistry in resected gastric cancer specimens showing focally positive (**G**) and invasive edge (**H**) patterns of staining.

Photomicrographs of HIF-1*α* staining in the different tissues are shown in Figure 1. Hypoxia-inducible factor-1*α* was not seen in biopsies of normal gastric mucosa, but expression increased in density and intensity with progression to gastric cancer (*P*=0.0001, Table 1). In the *H. pylori* associated gastritis biopsies, HIF-1*α* was expressed focally, with only a small percentage of weakly positive mucosal cells identified. The positive cells tended to be in small clusters or were part of the same crypt. Staining was predominantly in areas of inflammation associated with *H. pylori*. Ten of the 24 intestinal metaplastic biopsies expressed HIF-1*α*, and the intensity of staining was increased compared with the *H. pylori* associated gastritis, with one-third showing moderate nuclear staining. In the biopsies of intestinal metaplasia, HIF-1*α* was expressed predominantly in the metaplastic tissue areas. The nuclei of cells forming crypts containing goblet cells stained positively, whereas adjacent non-metaplastic crypts were negative. Hypoxia-inducible factor-1*α* was seen in half of the gastric dysplasia samples. Staining appeared to be more prevalent in high (four of six positive) *vs* low (two of six positive) grade lesions. The majority of intestinal-type adenocarcinoma specimens showed nuclear staining expression, with half staining strongly for HIF-1*α*. The highest percentages of expression were found in the diffuse-type adenocarcinoma biopsies.

### Expression of HIF-1*α* in surgically resected specimens

The predominant staining pattern observed in adenocarcinomas was focal in nature, with small numbers of positive cells adjacent to each other, rather than scattered single positive cells (Figure 1G). Individual malignant glands showed positive staining in either the majority or none of the cells. There was increased staining within superficial malignant cells in direct contact with the gastric lumen that did not appear artefactual. In the majority of cases, there was diffuse inflammation and necrosis of variable degree throughout the tumour with an associated desmoplastic reaction, as is often the case in gastric adenocarcinoma. It was, therefore, not possible to assess reliably staining patterns associated with inflammation and necrosis compared with non-inflammatory areas. However, it was noted that tumour cells adjacent to areas of surface ulceration that were peri-necrotic in nature showed increased levels of HIF-1*α* expression. Some of the tumours studied had large solid areas of malignant cells that showed no increase in HIF-1*α* expression within the central region of the cell groups but a tendency for increased expression in the peripheral layers of cells. Malignant cells at the invasive edge of the tumour tended to show increased staining that was more pronounced if the invasive edge was penetrating the subserosal tissue (Figure 1H). In some cases, the only positive tumour cells were those that had invaded through the muscularis propria into subserosal fat. It was interesting to note that macrophages and endothelial cells associated with tumour cells within the subserosal tissue also showed strong staining for HIF-1*α*. This feature was not seen in other layers of the gastric wall. In some cases, the adjacent non-neoplastic mucosa showed intestinal metaplasia that was associated with increased expression of HIF-1*α*, as seen in the biopsy specimens.

In addition to the predominantly nuclear expression, cytoplasmic staining was also observed, but was not scored. In 83 tumour sections (47%), no HIF-1*α* nuclear immunostaining was observed. Positive nuclear staining was as follows: <2% staining in 62 sections (35%), 2–10% staining in 21 sections (12%), 11–30% staining seven sections (4%) and >30% staining in three sections (2%). Staining pattern was focally positive in 49 (28%), at the invasive tumour edge in 41 (23%) and diffusely positive in three (2%). Staining intensity was similar between slides and not scored in the surgically resected specimens. One slide was lost after staining. All negative controls showed no immunoreactivity. Scoring was repeatable with good inter-observer agreement (*r*=0.90, *P*=0.0001).

### HIF-1*α* expression and clinicopathological features

For correlation with clinicopathological features, HIF-1*α* expression was categorised as negative (score 0) or positive (scores 1/2/3/4). Tables 2 and 3 summarise the distributions of patients according to tumour HIF-1*α* expression (positive *vs* negative) and staining pattern (negative, focal or invasive edge). Tumours that expressed HIF-1*α* tended to have a higher overall TNM stage compared with those that did not (*P*=0.045). There were no statistically significant differences between HIF-1*α* positive and negative tumours regarding differentiation, Lauren type, T stage, N stage or M stage (Table 2). When a higher cutoff was used to determine HIF-1*α* positivity (>2% staining), no statistically significant relationships were seen between high HIF-1*α* expression and clinicopathological features. There were no statistically significant differences between HIF-1*α* positive and negative tumours regarding differentiation, Lauren type, T stage, N stage or M stage (Table 2). Hypoxia-inducible factor-1*α* expression at the invasive edge was associated with lymph node metastases (*P*=0.034) and advanced TNM stage (*P*=0.001).

### HIF-1*α* expression and patient survival

**Figure 2:**
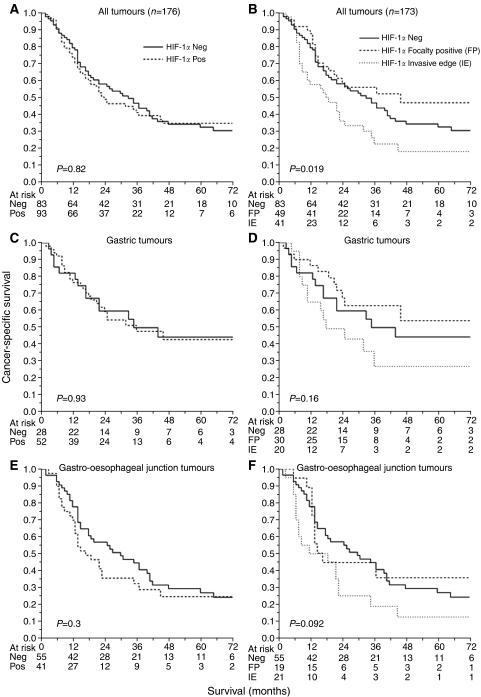
HIF-1*α* expression and patient outcome in patients with non-cardia gastric cancers or gastro-oesophageal junction tumours. Left hand column shows HIF-1*α* negative (score 0) *vs* positive (scores 1/2/3/4) expression (*n*=176). The right hand column shows HIF-1*α* expression pattern categorised as invasive edge, negative or focally positive (*n*=173; 3 tumours with diffuse expression were excluded). Log-rank *P*-values are given.

At the time of analysis, 51 patients were alive with a median follow-up of 48 months (range 13–118) months, whilst 107 had died of disease with a median time to death of 14 (range 2–74) months. There were 16 inter-current deaths from other causes. Table 4 summarises the results of univariate analyses of overall and cancer-specific survival. The results for HIF-1*α* expression in relation to cancer-specific survival are illustrated in Figure 2. There was no difference in overall and cancer-specific survival in patients with HIF-1*α* negative *vs* positive tumours, either for the group as a whole, when gastric and gastro-oesophageal junction tumours were analysed separately or when a higher cutoff (>2% staining) was used to determine positivity. However, in univariate analysis HIF-1*α* expression pattern was a significant prognostic factor for overall (*P*=0.016, log-rank test) and cancer-specific (*P*=0.019, log-rank test) survival. Using the Cox proportional hazards model and in comparison with tumours not expressing HIF-1*α*, invasive edge staining was associated with a hazard ratio of 1.6 (95% CI 1.0–2.5) and focally positive staining a hazard ratio of 0.7 (95% CI 0.5–1.2). Other significant factors in univariate analyses were tumour differentiation, T stage, N stage, overall TNM stage and R classification (Table 4). In multivariate analysis, only overall TNM stage and R classification retained prognostic significance for overall and cancer-specific survival. For example, R1/2 compared with R0 resections had a hazard ratio for cancer-specific survival of 2.0 (95% CI 1.2–3.2; *P*=0.006) and TNM stage 4 *vs* 1 disease was associated with a hazard ratio of 4.5 (95% CI 1.8–11.1; *P*=0.001).

## DISCUSSION

Hypoxia-inducible factor-1*α* expression increased in density and intensity with progression from normal mucosa to gastric cancer. This finding is consistent with other studies showing HIF-1*α* was not expressed in normal tissue but seen at an increasing level during the pathological process of cancer development, progression and loss of differentiation (Zhong *et al*, 1999), and that staining increased in breast (Bos *et al*, 2001), skin (Costa *et al*, 2001) and cervical (Acs *et al*, 2003) carcinogenesis. We studied HIF-1*α* expression in gastric biopsies corresponding with the proposed sequence of gastric carcinogenesis. It must be emphasised, however, that this model does not apply to gastro-oesophageal junction tumours. Gastro-oesophageal adenocarcinomas (Siewert Type I and II tumours) arise via a similar sequence of histopathological events, however, the initiating, promoting and molecular factors are different to gastric cancer carcinogenesis (Jankowski *et al*, 2000). Indeed, *H. pylori* appears to exert a protective role in these types of tumours (Chow *et al*, 1998).

The molecular mechanisms of gastric cancer development are unknown. Infection with *H. pylori* is the major initiating and driving factor, with the proposal that the formation of ROS owing to neutrophil infiltration in response to *H. pylori* infection causes epithelial cell injury and progressive DNA damage (Obst *et al*, 2000). In addition to ROS, another important mediator in the chronic inflammatory process is nitric oxide (NO), which, in response to *H. pylori* infection, is produced by gastric epithelial and non-epithelial cells from L-arginine via inducible-nitric oxide synthase (iNOS). Increased iNOS expression is seen in *H. pylori* infected gastric mucosa (Mannick *et al*, 1996; Pignatelli *et al*, 1998). A study also observed increased VEGF expression and new microvessel formation in *H. pylori* infected gastric mucosa (Tuccillo *et al*, 2005). Our observation that HIF-1*α* expression is an early feature of gastric carcinogenesis suggests it may play a role in promoting molecular changes that drive tumour formation. As the normal architecture and vascular supply of gastritis-associated mucosa and intestinal metaplasia is presumably maintained, HIF-1*α* expression in these specimens is probably not related to cellular hypoxia, but to inflammatory processes. In support of this idea, a recent cell line study showed the non-hypoxic stabilisation of HIF-1*α* by ROS produced from *H. pylori* (Park *et al*, 2003). Also, NO was shown to interfere with HIF-1*α* prolyl hydroxylases under normoxia, preventing degradation and resulting in HIF-1*α* accumulation and activation (Metzen *et al*, 2003). It may be, therefore, that in distal intestinal gastric cancer development *H. pylori* induced ROS and NO lead to HIF-1*α* stabilisation, which then plays a role in the stimulation of cell proliferation, protection from apoptosis and other molecular changes important in driving tumorigenesis. The observation that *H. pylori* infection induces gastric dysplasia in TP53 knockout but not wild-type mice highlights the obvious importance of genetic changes in tumorigenesis (Fenoglio-Preiser *et al*, 2003). Nevertheless, a recent paper showed that HIF-1*α* induces genetic instability and ‘provided molecular insights into the mechanisms underlying hypoxia-induced genetic instability’ (Koshiji *et al*, 2005). It may be, therefore, that HIF-1*α* is involved in a process of inflammation-mediated genetic instability.

Hypoxia-inducible factor-1*α* expression (positive *vs* negative) had no prognostic significance in patients with surgically treated gastric and gastro-oesophageal junction adenocarcinoma. Our finding agrees with a study of 146 Japanese patients, mainly with distal gastric cancer (Urano *et al*, 2006), and with some published reports showing HIF-1*α* has no prognostic significance in cervical (Haugland *et al*, 2002; Hutchison *et al*, 2004), colorectal (Yoshimura *et al*, 2004) and ovarian (Birner *et al*, 2001) cancers. In contrast, two further studies in gastric cancer showed HIF-1*α* expression was an adverse prognostic factor in multivariate analyses (Mizokami *et al*, 2006; Sumiyoshi *et al*, 2006). Hypoxia-inducible factor-1*α* was associated with poor clinicopathological features, VEGF expression and microvessel invasion (Mizokami *et al*, 2006). The combination of HIF-1*α* and non-functional TP53 expression indicated an extremely poor prognosis (Sumiyoshi *et al*, 2006).

In our study, two predominant types of HIF-1*α* expression were observed: staining of the tumour's invasive edge and focally positive staining. Hypoxia-inducible factor-1*α* staining of the invasive edge was associated with aggressive tumour characteristics such as lymph node metastases and worse overall stage. Other studies have noted HIF-1*α* expression at the invasive edge of tumours (Zhong *et al*, 1999; Zagzag *et al*, 2000), but have not performed survival analyses. In our analyses tumours with invasive edge staining had a worse prognosis compared with either HIF-1*α* negative or focal expression. Similarly the HIF-1*α* regulated CA9 was expressed at the invasive tumour edge in a subset of gastric cancer (Chen *et al*, 2005a) where it was associated with tumour invasion, advanced disease and a poor prognosis. Other authors showed that different patterns of HIF-1*α* expression were associated with different survival characteristics (Vleugel *et al*, 2005). In a study in breast cancer (Vleugel *et al*, 2005), peri-necrotic HIF-1*α* was associated with the expression of CA9 and Glut-1 and a poor prognosis. However, the diffuse staining type had a more favourable prognosis and was not associated with CA9 or Glut-1 expression.

We found that focally positive HIF-1*α* expression was associated with a less aggressive tumour phenotype and an improved prognosis. Some studies have found that HIF-1*α* expression in head and neck (Beasley *et al*, 2002), non-small cell lung (Volm and Koomagi, 2000) and renal cell (Lidgren *et al*, 2005) cancer is associated with an improved survival. However, as described in the Introduction, most studies have shown HIF-1*α* expression is associated with a poor prognosis (Griffiths *et al*, 2005). It has been suggested that HIF-1*α* expression may be a less important prognostic factor in surgically treated patients as the major influence of hypoxia-induced radiation resistance is lacking (Beasley *et al*, 2002). However, studies in patients with cervical cancer who underwent radiotherapy showed either a trend towards improved prognosis (Mayer *et al*, 2004) or an improved prognosis in a subgroup of patients (Hutchison *et al*, 2004). Although differences in staining and scoring methods cannot be ruled out completely, differences in prognostic outcome observed in numerous studies may reflect the differential regulation by HIF-1*α* of a range of downstream target molecules. This differential regulation might also be determined in individual tumours by the different processes leading to HIF-1*α* stabilisation (e.g. hypoxia/oncogene/ROS).

Hypoxia-inducible factor-1*α* can have both pro- and antiapoptotic effects (Piret *et al*, 2002), and can also both stimulate (Carmeliet *et al*, 1998) and inhibit (Bacon and Harris, 2004) proliferation. There is evidence for communication between HIF-1*α* and p53; p53 can stabilise HIF-1*α* and vice versa (Greijer and van der Wall, 2004; Schmid *et al*, 2004). Also, HIF-1*α* phosphorylation status may determine whether it acts to promote or check tumour cell survival. Dephosphorylated HIF-1*α* stabilised p53 and induced apoptosis, whereas phosphorylated HIF-1*α* bound to HIF-1*β* to form the HIF-1 transcription factor thereby promoting tumour growth (Suzuki *et al*, 2001). There is likely to be an intricate balance between the different roles of HIF-1*α*, which might be determined by the cumulative effect of multiple interactions within a cell. In the series of gastric cancer patients studied here, therefore, the beneficial effect of a focal pattern of HIF-1*α* expression on prognosis may relate to its proapoptotic and antiproliferative.

A final consideration that might play a role in determining whether HIF-1*α* expression is a good or bad prognostic factor is any contribution from other members of the HIF family. There are two other homologues of the *α*-subunit (HIF-2*α* and HIF-3*α*), which have different downstream actions and prognostic effects. A recent study showed that HIF-1*α* and HIF-2*α* upregulate different genes (Wang *et al*, 2005). Studies in non-small cell lung cancer and malignant melanomas showed that HIF-2*α* expression was related to a poor outcome when HIF-1*α* was not (Giatromanolaki *et al*, 2001, 2003). These findings raise the possibility of tissue specific differences in the relative importance of HIF proteins in determining tumour progression and prognosis. The clinical relevance of different HIF proteins and variants, therefore, will be of interest for future research in gastric cancer.

In conclusion, HIF-1*α* expression is an early event in gastric carcinogenesis and is apparent in specimens infected by *H. pylori*. Gastric biopsy specimens of intestinal metaplasia, dysplasia and intestinal type adenocarcinoma show a progressively increased density and intensity of HIF-1*α* staining. The prognostic impact of HIF-1*α* expression in gastric cancer appears to be dependent on the staining pattern with HIF-1*α* expression at the invasive tumour edge associated with a poor prognosis and focally positive expression a better prognosis. This relationship with a good outcome might be related to the proapoptotic and antiproliferative effects of HIF-1*α*. We hypothesise that variation in survival associated with different staining patterns may be related to the differential regulation by HIF-1*α* of a range of downstream target molecules.

## Figures and Tables

**Table 1:** HIF-1*α* expression in the gastric cancer progression sequence

		**HIF-1*α* density**					**HIF-1*α* intensity**		
**Biopsy type**	***n***	**0%**	**<2%**	**2–10%**	**11–30%**	**>30%**	**Weak**	**Mod**	**Strong**
Normal mucosa	20	20	—	—	—	—	—	—	—
*H. pylori* gastritis	24^a^	12	12	—	—	—	11	1	—
Intestinal metaplasia	24^a^	14	8	2	—	—	7	3	—
Dysplasia	12	6	2	3	1	—	1	2	3
Intestinal type adeno	19	3	7	5	3	1	6	2	8
Diffuse type adeno	21	11	3	2	2	3	1	0	9

Abbreviation: HIF=hypoxia inducible factor.

a Four biopsies had areas of *H. pylori* gastritis and intestinal metasplasia and were included in both subcategories.

The increases in HIF-1*α* density (*P*=0.0001) and intensity (*P*=0.0001) were highly statistically significant (Jonckheere–Terpstra test).

**Table 2:** Distribution of 176^a^ patients according to tumour HIF-1*α* expression

	**HIF-1*α* expression**		
**Factor**	**Negative**	**Positive**	*P* *
*Differentiation*			
Well	12	6	
Mod	33	34	
Poor	38	53	0.14
			
*Lauren type*			
Diffuse	41	50	
Intestinal	42	43	0.56
			
*T stage*			
T *in situ*	2	1	
T1	6	10	
T2	29	25	
T3	43	56	
T4	3	1	0.44
			
*N stage*			
N0	23	29	
N1	51	49	
N2	7	13	
N3	2	2	0.58
			
*M stage*			
M0	81	91	
M1	2	2	0.91
			
*Overall TNM stage*			
0	2	1	
I	11	20	
II	33	21	
III	31	48	
IV	6	3	0.045

Abbreviations: HIF=hypoxia inducible factor; TNM=tumour node metastasis.

a One patient with a missing slide was excluded.

* *χ*^2^ *P*-value.

**Table 3:** Distribution of 173^a^ patients according to tumour HIF-1*α* staining pattern

	**Pattern of HIF-1*α* staining**			
**Factor**	**HIF-1*α* focal positivity**	**HIF-1*α* negative**	**HIF-1*α* at the invasive edge**	***P*** *
*Differentiation*				
Well	4	12	2	
Mod	20	33	14	
Poor	25	38	25	0.37
				
*Lauren type*				
Diffuse	24	41	23	
Intestinal	25	42	18	0.74
				
*T Stage*				
T *in situ*	1	2	0	
T1	7	6	3	
T2	18	29	6	
T3	22	43	32	
T4	1	3	0	0.087
				
*N Stage*				
N0	22	23	5	
N1	20	51	28	
N2	6	7	7	
N3	1	2	1	0.034
				
*M stage*				
M0	48	81	40	
M1	1	2	1	0.99
				
*Overall TNM stage*				
0	1	2	0	
I	16	11	3	
II	13	33	7	
III	17	31	30	
IV	2	6	6	0.001

Abbreviations: HIF=hypoxia inducible factor; TNM=tumour node metastasis.

a Three patients with diffusely positive HIF-1*α* staining and one patient with a missing slide were excluded.

* *χ*^2^ *P*-value.

**Table 4:** Univariate survival analysis of putative prognostic factors following surgical resection for gastric and gastro-oesophageal cancer

	**Overall survival**			**Cancer-specific survival**		
**Parameter**	**HR**	**95% CI**	***P*** ^*^	**HR**	**95% CI**	***P*** ^*^
*HIF*						
0	1	—	—	1	—	
1/2/3/4	1.1	0.8–1.4	0.62	1.0	0.7–1.5	0.82
						
*HIF*						
Negative	1	—	—	1	—	—
Focal	0.9	0.5–1.3	0.49	0.7	0.5–1.2	0.26
Invasive edge	1.6	1.0–2.4	0.042	1.6	1.0–2.5	0.047
						
*Diff*						
Well	1	—	—	1	—	—
Mod	2.9	1.4–6.2	0.005	3.4	1.3–8.5	0.011
Poor	3.7	1.8–7.8	0.001	5.3	2.1–13.3	0.001
						
*Lauren type*						
Intestinal	1	—	—	1	—	—
Diffuse	1.4	1.0–2.0	0.052	1.8	1.2–2.6	0.003
						
*Location*						
Non-GOJ	1	—	—	1	—	—
GOJ	1.4	1.0–2.0	0.083	1.5	1.0–2.2	0.059
						
*T stage*						
T0/1	1	—	—	1	—	—
T2	2.6	1.0–6.7	0.052	5.2	1.2–22.0	0.023
T3	4.8	1.9–12.0	0.001	9.6	2.3–39.0	0.002
T4	16.8	4.4–64.2	0.0001	37.5	6.8–207.6	0.0001
						
*N stage*						
N0	1	—	—	1	—	—
N1	2.0	1.3–3.0	0.003	2.5	1.5–4.1	0.001
N2	3.5	1.9–6.4	0.0001	4.8	2.5–9.2	0.0001
N3	4.2	1.5–12.0	0.008	5.7	1.9–16.9	0.002
						
*M stage*						
M0	1	—	—	1	—	—
M1	2.6	1.0–7.1	0.062	2.9	1.1–7.9	0.037
						
*Overall TNM stage*						
0/1	1	—	—	1	—	—
2	1.4	0.8–2.6	0.25	1.8	0.9–3.6	0.12
3	3.3	1.9–5.9	0.0001	4.5	2.3–8.8	0.0001
4	7.6	3.3–17.5	0.0001	10.9	4.4–27.1	0.0001
						
*R class*						
R0	1	—	—	1	—	—
R1	2.3	1.6–3.3	0.0001	2.7	1.8–4.0	0.0001
R2	5.8	2.9–11.6	0.0001	7.2	3.6–14.5	0.0001

Abbreviations: CI=confidence interval; HR=hazard ratio; TNM=tumour node metastasis. ^*^Obtained using a univariate Cox-proportional hazards model.
